# Supplementary material for: Rapid turnover of life-cycle-related genes in the brown algae
Source: Genome Biol. 2019 Feb 14;20:35. doi: 10.1186/s13059-019-1630-6 (PMC6374913; doi:10.1186/s13059-019-1630-6)
Supplement: Supplementary file 3 — Protein alignments, corresponding Gblocks html file and CDS sequences for the lineage conserved gametophyte-biased genes. (ZIP 2109 kb) [file 13059_2019_1630_MOESM3_ESM.zip › Con_GA_alignments/Ec-26_003000.aa_ali.fasta-gb.htm]

Ec-26\_003000.aa.fasta.trans.aa\_ali.fasta


## Gblocks 0.91b Results

Processed file: **Ec-26\_003000.aa.fasta.trans.aa\_ali.fasta**  
Number of sequences: **2**  
Alignment assumed to be: **Protein**  
New number of positions: **1022** (selected positions are underlined in blue)

```
                         10        20        30        40        50        60
                 =========+=========+=========+=========+=========+=========+
Ec-26_003000     MGPKKKAAKAAAAPAKAAAAPAGGAAGPGVGGTETSTAATFTPAEAKKLAKLLKASDRGK
MSTRG.10931.1    ------------------------------------------------------------
                                                                             


                         70        80        90       100       110       120
                 =========+=========+=========+=========+=========+=========+
Ec-26_003000     RLDAIGVVSENHRYIASGGCLLPLLESVVAKKKMEDVVLAASQVIVDYLVGRDPGGDPTV
MSTRG.10931.1    ------------------------------------------------------------
                                                                             


                        130       140       150       160       170       180
                 =========+=========+=========+=========+=========+=========+
Ec-26_003000     VTDSAMAALQEAKKKPAKYKLAEVLTNLVLNPDQQNSWNAVRSLRSIAAAVHSLPAGSSQ
MSTRG.10931.1    ------------------------------------------------------------
                                                                             


                        190       200       210       220       230       240
                 =========+=========+=========+=========+=========+=========+
Ec-26_003000     HASLGAMLLKPGKPVERLCGYLVAQEAQQSTPVETPEAKKGAAAEGRDPVVPWPVVRLQV
MSTRG.10931.1    ------------------------------------------------------------
                                                                             


                        250       260       270       280       290       300
                 =========+=========+=========+=========+=========+=========+
Ec-26_003000     MDTIGVMCGVSDEAVQTVVSTGTVRCVLGVLRSITESEAPDMALLTSTLVILFAVAKAGG
MSTRG.10931.1    ------------------------------------------------------------
                                                                             


                        310       320       330       340       350       360
                 =========+=========+=========+=========+=========+=========+
Ec-26_003000     GGIAPEVGIAEGDIASGSSPGVAMAATAGVGASSGGEMPSELAALAEGGAWGAVYDALKG
MSTRG.10931.1    ------------------------------------------------------------
                                                                             


                        370       380       390       400       410       420
                 =========+=========+=========+=========+=========+=========+
Ec-26_003000     PCMARATELQQASATAGGAKKGAADAAAAVSADLTGCLDNMARCAEVLRVMAMTGAGLEE
MSTRG.10931.1    ------------------------------------------------------------
                                                                             


                        430       440       450       460       470       480
                 =========+=========+=========+=========+=========+=========+
Ec-26_003000     EGALEAGVAAMSSILGMEGLPREQAAVANLMTGMVKAMGSIACLSESNRIKVCSAGAAAK
MSTRG.10931.1    ------------------------------------------------------------
                                                                             


                        490       500       510       520       530       540
                 =========+=========+=========+=========+=========+=========+
Ec-26_003000     VLALMAPPDPSASGVGGSSSPPDTPHGRLTRKTAEKCLHHLVVSRVTWLADDRALCDTSA
MSTRG.10931.1    ------------------------------------------------------------
                                                                             


                        550       560       570       580       590       600
                 =========+=========+=========+=========+=========+=========+
Ec-26_003000     PPADTPESSQDIPPPSGNAAGAGAGEGGDNEKFGTAETASRPRRSGSFAAAAAPNDPPPA
MSTRG.10931.1    ------------------------------------------------------------
                                                                             


                        610       620       630       640       650       660
                 =========+=========+=========+=========+=========+=========+
Ec-26_003000     LGPAYLSVDAVLAAATAGSIDVRCRAIRLLSRLMSSPRCASALGVSAVPALGRLIEWWLQ
MSTRG.10931.1    ------------------------------------------------------------
                                                                             


                        670       680       690       700       710       720
                 =========+=========+=========+=========+=========+=========+
Ec-26_003000     PKNGPEAQRTPRPSEPAASEGGGGKSAAGAAAAGGGKKGAGGKAGAKEAAAAAAATAAAE
MSTRG.10931.1    ------------------------------------------------------------
                                                                             


                        730       740       750       760       770       780
                 =========+=========+=========+=========+=========+=========+
Ec-26_003000     AASKNEQAAKDEVAAAAAARAAAKELEESDSTRVLRDEALAYALTVMLKLAEAGREQRAA
MSTRG.10931.1    ---------------------AAKKLEESDSTRVLRDEALAYALTVMLKLAESGGEERAA
                                      #######################################


                        790       800       810       820       830       840
                 =========+=========+=========+=========+=========+=========+
Ec-26_003000     IGENATVELLAGVLKSLPCTPEEFYANGGEHLGSMTVCAAVAAGTARGTKSPTIPAAAAV
MSTRG.10931.1    IGEVSVVELLVSVLKMLPCTPEEFYANAGEKRRSMTGSGVAGVGGSAAPPVSTTPALAP-
                 ##########################################################  


                        850       860       870       880       890       900
                 =========+=========+=========+=========+=========+=========+
Ec-26_003000     DEPAVVTADNGGGNIGTHHAPSHAADDSLLAAPKPDTTLDLSLAHRRPSSGGNPRKLCCW
MSTRG.10931.1    ---AGKAGEKGLGSGDALHAPSHAADGGLLN-PKLDTTLDLSLAQHRPGSSNTPRKLCCW
                    ###########################  ############################


                        910       920       930       940       950       960
                 =========+=========+=========+=========+=========+=========+
Ec-26_003000     RGAKSRDPEVPLFSPLDWGWDFEVGVSQVPVQPGLVLRAAALRVLLAVVDGYDPAVEATA
MSTRG.10931.1    RGEKSRDPEVPLFSPLDWGWDFQVGVSQEPIQPGLVLRAAALRVLLAVADGYDPAAEAVA
                 ############################################################


                        970       980       990      1000      1010      1020
                 =========+=========+=========+=========+=========+=========+
Ec-26_003000     AAEAAASAPAAGSKGGAGAAAAAAAETSAKAAALSGGSGARTVLKHVLPVCLDLLSVDVR
MSTRG.10931.1    AAGAASTAPATKG----GAAAAAAADTSAKAAALSGGKGARSVLSRALPVCVDLLSVDVR
                 ##########       ###########################################


                       1030      1040      1050      1060      1070      1080
                 =========+=========+=========+=========+=========+=========+
Ec-26_003000     HAGRDDDGVEGENRGGGSCSSRNHGESGTEADRKTASQPKEQQQQQQQQGQDADNAGGQS
MSTRG.10931.1    HAR------------------------------------------------EIGEAGGQP
                 ##                                                     #####


                       1090      1100      1110      1120      1130      1140
                 =========+=========+=========+=========+=========+=========+
Ec-26_003000     FNSSLSADFLSGRPVSPSEELVHKEIRVACLRLLGSLLRLGGTAREGFLSTSATHRDGGF
MSTRG.10931.1    PIDKPTADDLSGWPVSPSEELVHEEIRVACLRLLGSLLRLGGVAREALLSVAATHRGGGF
                 ############################################################


                       1150      1160      1170      1180      1190      1200
                 =========+=========+=========+=========+=========+=========+
Ec-26_003000     THIKELCGSPREEKEETAPPARK---TTGAAAAEKNNAGGENAG----GWVRPESFRSWN
MSTRG.10931.1    KHLEEHSGRSCTNADAQAPTSTKNTGTTGDRGTAAKNSGGEEVDGGVSPWVKPDSFRDWN
                 ########                  ###############        ###########


                       1210      1220      1230      1240      1250      1260
                 =========+=========+=========+=========+=========+=========+
Ec-26_003000     LSAGEGCEPASLRDSLPYVRMISMFMLPLRNPDAPITDIVAALVALKRLCRENEHETGGT
MSTRG.10931.1    LRG--ECDLNSIRASLPYARALSMFLLPLRNPDAPATDIVAALVAMKRLCREDEHEAGGT
                 #     ######################################################


                       1270      1280      1290      1300      1310      1320
                 =========+=========+=========+=========+=========+=========+
Ec-26_003000     QPEPLPQSAENDESVPPLPSSREGTAGVLVDTIAGVAVSMGALVPLMSIWGCALAAAGSA
MSTRG.10931.1    QPEAHPPSAEDDQ-IPHLPSCREGTAGALVDTLAGVAVGMGALVPLVSIWGCAVAAAGPA
                 ############   #############################################


                       1330      1340      1350      1360      1370      1380
                 =========+=========+=========+=========+=========+=========+
Ec-26_003000     ADLAPEETGMVNECQALIDYLIRRGHAREEFWSSLPSLGQIAETKAAAAEAAAPKKAPRK
MSTRG.10931.1    VDLAPGITGMVSECQGLINYLIRRGHAREDFWSSLPSPEQISEARAAAADSAAPRKAPRK
                 ############################################################


                       1390      1400      1410      1420      1430      1440
                 =========+=========+=========+=========+=========+=========+
Ec-26_003000     SKGSAVKAGKGGGGDGGKGSR-SSVTGLEVEDEEKPDAAPKPPEPPAGRPDPNLGPNRAS
MSTRG.10931.1    SKGSSGKAGKGGG----KGSRDPSTAALETEEEEMPD-EPKPPDPPAGRPDPNMGPDRAS
                 #############          ##############  #####################


                       1450      1460      1470      1480      1490      1500
                 =========+=========+=========+=========+=========+=========+
Ec-26_003000     WSKLLDSRMDEQRTQTCGTTALLMATVTGLETAVGNLLLAGADPNVRGKDGRSPLMCALA
MSTRG.10931.1    WGQLLNARVDEQRTQTRGTTALLMATVTGLETAVGNLLLAGADPNVRGCDGRTPLMCALA
                 ############################################################


                       1510      1520      1530      1540      1550      1560
                 =========+=========+=========+=========+=========+=========+
Ec-26_003000     QGMDEAAHGLVAAGADVDAVNHLGDNVLKCAFLCPSRQAMRDVMRKGPEAAGAEAVPMAP
MSTRG.10931.1    QGMDEAARGLVEAGADVDAVNLEGDSVLRCAFLCPARRTMRRVMRKRPETRE--------
                 #################################################           


                       1570      1580      1590      1600      1610      1620
                 =========+=========+=========+=========+=========+=========+
Ec-26_003000     SSGWAGPLSSASSSRRGSTAPSIDGWSG-RVGRRRSNS---RSVSRSRSRSRSRSGSLTR
MSTRG.10931.1    -DG-ATP-GSAGSGRRGLTTPIDGGFNGGQVGRRQSSSSHGMSRSGSRSRSRSRSGSLTR
                          ###################              ##################


                       1630      1640      1650      1660      1670      1680
                 =========+=========+=========+=========+=========+=========+
Ec-26_003000     DRRRSSLGRSVSFGEGAFGFDTAGGAASSGGSGGGRRRVSRMASLSALDSARAALHDLAP
MSTRG.10931.1    DRRRSSLSRSISFGEEVLVGDTAVAAASGTTGGAHGRRLSRMASLSALDSAREALRESAP
                 ############################################################


                       1690      1700      1710      1720      1730      1740
                 =========+=========+=========+=========+=========+=========+
Ec-26_003000     RESARPLKTPRGTTIVRGDARMVPYILGCGADPNVSSASGDFPLHWAVSGTELTVKIMNQ
MSTRG.10931.1    RESARPLKTPRGTTVVPGDARMVPYILECGADPNVSSGSGEFPLHWAVTGAELTVRIMNQ
                 ############################################################


                       1750      1760      1770      1780      1790      1800
                 =========+=========+=========+=========+=========+=========+
Ec-26_003000     RVKIVAGGTDGGDGVVRGEGHGKTTRADVGEDSSSNGSNANRPAAAAAGATVAGAAATEQ
MSTRG.10931.1    KVKIVAAGGGGGS----GDCGGETTQVARKETAMSGEDGSVDIANPSTRTEIA-------
                 ############     ####################################       


                       1810      1820      1830      1840      1850      1860
                 =========+=========+=========+=========+=========+=========+
Ec-26_003000     GAAEHEQDVALLKVLVGAGSALDACNPDGMTALHAAVLAGRGTLAGTLLDAGASPNYSDS
MSTRG.10931.1    -GDGHQGDLALLEVLIRAGSALDACNPEGMTALHTAVIAGRESLAETLLDAGASPNVSDS
                     ########################################################


                       1870      1880      1890      1900      1910      1920
                 =========+=========+=========+=========+=========+=========+
Ec-26_003000     LGCLPLHYACLRAVAGYADLANRLLALGMGRPLDKGVHRDLRKGKTRREKLILDVADIMH
MSTRG.10931.1    LGCLPLHYACLRATAGYADLASRLLALGMGRPFNAGVYRDLRKGKTRREKLLLDVADIMH
                 ############################################################


                       1930      1940      1950      1960      1970      1980
                 =========+=========+=========+=========+=========+=========+
Ec-26_003000     KGLREATEPSSITQHRATRSELLNFVSAEGLTPIHYVCDGRIVGRHAAAAVLALWEERAP
MSTRG.10931.1    KGLCEAX-----------------------------------------------------
                 ######                                                      


                       1990      2000      2010      2020      2030      2040
                 =========+=========+=========+=========+=========+=========+
Ec-26_003000     PATAAPGTTILNGGGPDSGGGEGRVTVEDGSIDRVKTLRWLLSESEVDPTVRLPRGATTL
MSTRG.10931.1    ------------------------------------------------------------
                                                                             


                       2050      2060      2070      2080      2090      2100
                 =========+=========+=========+=========+=========+=========+
Ec-26_003000     HLASRTPGSQGADLVRVLTHAGGVSLDTLDSPAGAMVGLTGQTASANGPSDADGGSPPLR
MSTRG.10931.1    ------------------------------------------------------------
                                                                             


                       2110      2120      2130      2140      2150      2160
                 =========+=========+=========+=========+=========+=========+
Ec-26_003000     FSALHYALQAGAWESARLLLSAGARVRPEGSFPPCLHVACLAGAPASLVKALLDGDSQAS
MSTRG.10931.1    ------------------------------------------------------------
                                                                             


                       2170      2180      2190      2200      2210      2220
                 =========+=========+=========+=========+=========+=========+
Ec-26_003000     STTILPAGAEPYTASPLLLAAASGNADLVEMLLSTAGGIGSIVEDHTAMLVGTGGDDGVP
MSTRG.10931.1    ------------------------------------------------------------
                                                                             


                       2230      2240      2250      2260      2270      2280
                 =========+=========+=========+=========+=========+=========+
Ec-26_003000     RTASAEGSESIWTMEHSPSDGRNPLHAAAAEGHMLAALVLVDADADATRDGSAPRSWLNT
MSTRG.10931.1    ------------------------------------------------------------
                                                                             


                       2290      2300      2310      2320      2330      2340
                 =========+=========+=========+=========+=========+=========+
Ec-26_003000     PDIEGNTPLDVAVYGGHWECAERLAVAERFDIRLAVERGPSSSLIVVERANMAIVDEGSG
MSTRG.10931.1    ------------------------------------------------------------
                                                                             


                       2350      2360      2370      2380      2390      2400
                 =========+=========+=========+=========+=========+=========+
Ec-26_003000     DPQTLRALRESNKLVMALLKRLHDTAGETVAAAAAATTTTRPRNGQGGGDRPDNRRKGMR
MSTRG.10931.1    ------------------------------------------------------------
                                                                             


                 
                 =====
Ec-26_003000     MIERR
MSTRG.10931.1    -----
```

```
Parameters used
Minimum Number Of Sequences For A Conserved Position: 2
Minimum Number Of Sequences For A Flanking Position: 2
Maximum Number Of Contiguous Nonconserved Positions: 8
Minimum Length Of A Block: 10
Allowed Gap Positions: None
Use Similarity Matrices: Yes
```

```
Flank positions of the 15 selected block(s)
Flanks: [742  838]  [844  870]  [873  970]  [978  1022]  [1076  1148]  [1167  1181]  [1190  1201]  [1207  1272]  [1276  1393]  [1404  1417]  [1420  1549]  [1570  1588]  [1603  1752]  [1758  1793]  [1805  1926]  

New number of positions in Ec-26_003000.aa.fasta.trans.aa_ali.fasta-gb:  1022  (42% of the original 2405 positions)
```
